# Nitric oxide-mediated thermomemory: a new perspective on plant heat stress resilience

**DOI:** 10.3389/fpls.2025.1525336

**Published:** 2025-02-28

**Authors:** Sheeba Naaz, Anjali Pande, Ashverya Laxmi

**Affiliations:** National Institute of Plant Genome Research, New Delhi, India

**Keywords:** epigenetic regulations, heat stress, heat shock proteins, nitric oxide, thermomemory

## Abstract

In the intricate world of plant responses to environmental stress, the concept of thermomemory has emerged as a fascinating and complex phenomenon. Plants, as sessile organisms, continually face the challenge of adapting to fluctuating climates, and the ability to “remember” prior heat stress encounters, a phenomenon known as thermomemory is a testament to their remarkable adaptability. Nitric oxide (NO), a versatile signaling molecule in plant physiology, has been implicated in a myriad of cellular processes crucial for stress adaptation. From its involvement in stomatal regulation to its influence on gene expression and antioxidant defense mechanisms, NO emerges as a central orchestrator in the plant’s response to elevated temperatures. Exploration of NO-mediated pathways provides insights into how plants not only cope with immediate heat stress but also retain a memory of these encounters. Unraveling the molecular intricacies of NO’s involvement in thermomemory enhances our understanding of the sophisticated strategies employed by plants to navigate a changing climate, offering potential avenues for innovative approaches to enhancing crop resilience and sustainable agriculture.

## 1 Introduction

As plants continually face the challenges posed by changing climatic conditions, understanding their adaptive strategies becomes imperative. Heat stress, a significant environmental threat, prompts plants to employ a repertoire of responses, with recent research highlighting the potential involvement of NO in the formation and retention of thermomemory (Seth and Sebastian, 2024). Heat is one of the biggest environmental challenges plants faces, and it forces them to adapt various strategies to mitigate its deleterious effects.

In plant systems, nitric oxide (NO) is a multipurpose gaseous signaling molecule involved in both physiological and pathological processes. It is now well known that NO plays a key role in signaling, plant growth, development, and stress responses. This adaptable molecule is involved in several developmental stages of plants, including seed germination, root growth, blooming, pollen tube growth, and leaf senescence. It also regulates stem cell activity, stomatal movement, primary metabolism, and defense against biotic and abiotic stressors (Tuteja et al., 2004; Pande et al., 2021). Although the plant science community is paying more and more attention to nitric oxide because of its role in resistance to different plant stress conditions, it is still unclear how NO affects a plant’s ability to withstand heat stress. Numerous lines of evidence point to NO as a crucial signaling molecule that mediates a range of plant responses, including gene expression, protein changes, oxidative defense, photosynthesis, and osmolyte accumulation in response to heat stress (Groß et al., 2013; Fancy et al., 2017). In addition, an increasing number of studies have revealed how NO interacts with phytohormones and other signaling molecules to achieve heat tolerance. For agriculture, one of the main abiotic stressors is high temperatures (Domingos et al., 2015). Heat stress results in cell injury and death by denaturing and inactivating many enzymes, damaging membrane proteins, and building up reactive oxygen species. Plants possess thermosensors that can identify particular changes and initiate protective mechanisms. According to reports, treatments with high temperatures cause different plant species to accumulate more NO (Yu et al., 2014). Although these results emphasize the emission of heat-induced NO as a critical response for enhancing plant adaptability to stress, NO release depends on the intensity and duration of heat treatment (Bouchard and Yamasaki, 2008; Yu et al., 2014).

Recent research suggests that nitric oxide (NO), a versatile signaling molecule in plant cells, may play a role in thermomemory, a phenomenon in which plants can’remember’ and adapt to previous heat stress exposure. This adds a layer of complexity to our understanding of plant response mechanisms (Parankusam et al., 2017; dos Santos et al., 2022).

This review explores the molecular pathways underlying heat-stress responses in plants, emphasizing the complex role that NO plays in determining thermomemory. By delving into the molecular intricacies of NO-mediated pathways, we seek to unravel the mechanisms through which plants not only endure immediate thermal challenges but also acquire the capacity to ‘memorize’ these experiences. In doing so, we aim to deepen our understanding of the adaptive strategies plants employ to thrive in the face of heat stress, offering insights that may pave the way for innovative solutions to enhance crop resilience and contribute to sustainable agricultural practices in a changing climate. Here, we explore current gaps in knowledge and highlight areas for future research, such as identifying specific NO-responsive elements and elucidating the temporal dynamics of NO-mediated thermomemory. The exploration of the role of nitric oxide (NO) in plant thermomemory against heat stress brings forth a complex scenario marked by challenges and unexplored avenues. One significant challenge lies in deciphering the specific molecular mechanisms through which NO contributes to the establishment and modulation of thermomemory. While recent researches suggest the involvement of NO, a comprehensive understanding of the intricate signaling pathways and downstream effects remains elusive. Unraveling the temporal dynamics of NO-mediated thermomemory is another challenge, as the duration and persistence of NO’s influence on plant memory retention require further elucidation. Furthermore, the interaction between NO and other signaling molecules in the context of thermomemory is a complex problem.

Investigating the synergies or antagonisms between NO and hormones, secondary messengers, or other signaling molecules is essential for a holistic understanding of plant responses to recurring heat stress events. Understanding how NO may impact gene expression to generate thermomemory is made possible by epigenetic modifications, which play a role in memory formation.

Epigenetic modifications are an important way for plants to generate thermomemory and control gene expression in response to heat stress. These alterations include DNA methylation, histone modifications, and short RNAs, which help to maintain long term stress-induced molecular changes. Similarly, heat stress can affect DNA methylation patterns, resulting in the stable repression or activation of particular thermotolerance-related genes. Small RNAs (miRNAs and siRNAs) play an important role in fine-tuning heat stress responses by modulating target gene expression after transcription (Li et al., 2024). According to studies, NO may influence these epigenetic pathways by changing chromatin-associated proteins via S-nitrosylation, affecting their stability and function (Corpas et al., 2015). Furthermore, NO may control the expression of epigenetic modifiers including histone deacetylases (HDACs) and DNA methyltransferases (DNMTs), influencing thermomemory formation (Hickok et al., 2013).

Furthermore, the translation of research findings into practical applications for crop improvement remains a challenge. Developing strategies to harness NO-mediated thermomemory for enhancing crop resilience in real-world agricultural settings requires bridging the gap between laboratory discoveries and field-level implementation.

## 2 Heat stress and thermomemory in plants

### 2.1 Heat stress

Heat stress (HS) is caused when plants are exposed to high temperatures for an extended period of time. As global temperatures continue to rise due to climate change, heat stress has become a significant factor impacting agricultural productivity and ecological balance. The plant’s developmental stage determines how HS affects plant productivity for instance, plants in their reproductive stage are extremely heat-sensitive which negatively affects anther development. (Resentini et al., 2023).

HS has a direct effect on the physiological and metabolic systems of plants, changing their typical gene expression patterns (Driedonks et al., 2015). The primary effects of HS on leaves are curling, necrosis, and senescence. Additionally, HS alters the fluidity of the membrane, which results in the plant cell’s electrolytic leakage (Savchenko et al., 2002). It also leads to the denaturation of proteins, including enzymes crucial for various metabolic processes. This leads to a breakdown in cellular functions, affecting energy metabolism, nutrient assimilation, and other essential pathways (Sachdev et al., 2021). Nearly 90% of the biomass produced on earth is a result of photosynthesis, a fundamental process for plant growth which is extremely vulnerable to heat stress. Photosystem-II (PS-II) is the most susceptible to damage, however several other proteins of chloroplast denature or cease to function at high temperatures (Tang et al., 2007). The structural integrity of the photosynthetic apparatus is compromised by heat stress, which limits the fixation of carbon dioxide (Ul Haq et al., 2019). Heat stress inhibits the activity of key enzymes involved in photosynthesis, reducing carbon assimilation, and potentially leading to diminished yields. HS damages the electron transport mechanisms in the mitochondria and chloroplasts. It generates reactive oxygen species (ROS) such as superoxides (O_2_^-^), hydrogen peroxide (H_2_O_2_), and hydroxyl radicals (OH-) that damage DNA and induce lipid peroxidation of the cell membrane (Choudhury et al., 2017). Lipid peroxidation, a process that damages membrane lipids, disrupts membrane structure and fluidity, impacting cellular stability and function (Sachdev et al., 2021). Through the lipase enzyme HIKESHI-including PROTEIN1 (HLP1), plants can liberate polyunsaturated fatty acids, such as alpha-linolenic acid (18:3), from chloroplast membrane glycerolipids, such as monogalactosyldiacylglycerol (MGDG) under heat stress (Higashi et al., 2018). Another study reported that two membrane proteins, PHOSPHOLIPASE D (PLD) and PHOSPHATIDYLINOSITOL PHOSPHATE KINASE (PIPK), are activated by a fast increase in temperature (Mishkind et al., 2009). PLD and PIPK use membrane lipids to produce phosphatidic acid (PA) and phosphatidylinositol 4,5-bisphosphate (PIP2), respectively (Mishkind et al., 2009). PIP2 serves as a precursor to IP3, or inositol 1,4,5-triphosphate, which raises the amount of calcium in the cytoplasm by further activating IP3-gated calcium channels. Calcium accumulation then determines the activation of Heat shock response genes and ROS generation as a result of heat stress.

Heat stress has severe consequences on the reproductive development of plants. It can result in flower abortion, reduced pollen viability, and impaired seed formation, leading to decreased crop yields and economic losses in agriculture (Resentini et al., 2023).

Plants have several complex defense mechanisms to cope with atmospheric heat. The key players in this process are the heat shock proteins (HSPs) (Baniwal et al., 2007; Driedonks et al., 2015; Ul Haq et al., 2019). HSPs serve as molecular chaperones that stop the denaturation and aggregation of proteins. Moreover, HSPs interact with heat shock factors (HSFs) and modify their roles. HSFs activate numerous genes, including HSPs, through transcription.

Another way through which plants respond to heat stress is by adjusting the opening and closing of stomata. Stomatal closure is a protective mechanism to reduce water loss through transpiration, though it can also hinder the uptake of carbon dioxide for photosynthesis.

Plants respond to heat stress through genetic and metabolic adjustments. Metabolic pathways may also be altered to cope with the increased energy demands and cellular damage. Different plant species and cultivars exhibit varying sensitivities to heat stress. Some plants have developed specific mechanisms for heat tolerance, while others may be more susceptible. Understanding this variability is crucial for breeding and selecting heat-tolerant crops. Plants can undergo long-term adaptation and acclimation to recurrent heat stress events. This process involves changes in gene expression, adjustments in metabolic pathways, and the development of thermotolerance mechanisms to enhance resilience over time (Liu et al., 2006).

### 2.2 Thermomemory

In the face of rising global temperatures and climate uncertainties, plants exhibit a sophisticated adaptive strategy that goes beyond immediate stress responses. Thermomemory represents the remarkable ability of plants to retain information about past heat stress events and subsequently modulate their responses for enhanced resilience. Plants develop thermotolerance after being exposed to high heat for a short period, this process, called “priming,” helps them withstand extreme temperatures later on. As they adjust to the new, higher temperatures, they undergo “acclimatization,” which allows them to maintain stable growth and development. This adjustment helps them survive longer under prolonged heat stress conditions. Such enhanced heat tolerance mediated by thermomemory is commonly known as thermally acquired tolerance (TAT). Primed plants exhibiting TAT, which aids in their recovery, respond efficiently and robustly to subsequent exposure to HS conditions. Moreover, it is well-known that thermomemory remains somatically preserved in plants within a generation (Nishad and Nandi, 2021).

Recent research has unveiled intriguing molecular and physiological mechanisms underlying thermomemory, suggesting that plants possess a form of thermal ‘memory’ that enables them to mount more efficient and robust defenses upon subsequent exposures to elevated temperatures. This phenomenon involves intricate signaling pathways, potential epigenetic modifications, and the induction of specific stress-related genes (Zhao et al., 2020).

Different types of thermosensors like CNGC, ELF3, Ca^++^, glucose and PIF7 are present in plants (Dai Vu et al., 2019; Wu et al., 2023). Plant cellular components, such as the plasma membrane, nucleic acid, and proteins, respond to heat shock primarily through protein denaturation, nucleic acid fragmentation, and membrane fluidity—all of which may function as heat sensors (Dai Vu et al., 2019; Sharma et al., 2020). Phytochrome B is a red-light photoreceptor that has been involved in thermosensory properties in addition to its known light signaling role. Red light is absorbed by the dormant Pr form of phytochrome, which transforms into the active Pfr form, which then absorbs far-red light to return to Pr. High temperatures accelerate the conversion of Pfr to Pr form (Rockwell and Lagarias, 2006; Dai Vu et al., 2019). One study reported that Phytochrome interacting factor 4 (PIF4) and other PhyB signaling components participate in thermosensing (Kumar et al., 2012) Another potential plant thermosensor is the Physcomitrella patens, Cyclic Nucleotide Gated Calcium Channel B (CNGCb) or its orthologue, CNGC2, which is present in Arabidopsis was also reported. According to a different study, plants use the CNGCb and CNGC2 components of the Ca^++^ channel, which is cyclic nucleotide-gated in the plasma membrane, as their primary thermosensors. When this channel is disrupted, plants exhibit markedly elevated heat sensitivity (Finka et al., 2012). Mild heat stress causes heat sensors to become active, which may set off a chain reaction that activates many transcriptional regulators and changes the expression of a stress-related protein to provide resistance. Another study stated that Heat-shock proteins and other stress-responsive genes are essential for the development of heat-stress memory, and their expression could indicate a structural foundation due to calcium-dependent modulation (Zheng et al., 2024).

Additionally, Homolog D of respiratory burst oxidase (RBOHD), which is expressed throughout all tissues and serves as the primary catalyst for ROS signaling in plants, is activated by this downstream signaling. Superoxide is produced in the apoplastic region by RBOHD, a calcium-dependent NADPH oxidase, in response to various stressors, such as heat and pathogens (Qi et al., 2018).

**Figure 1:**
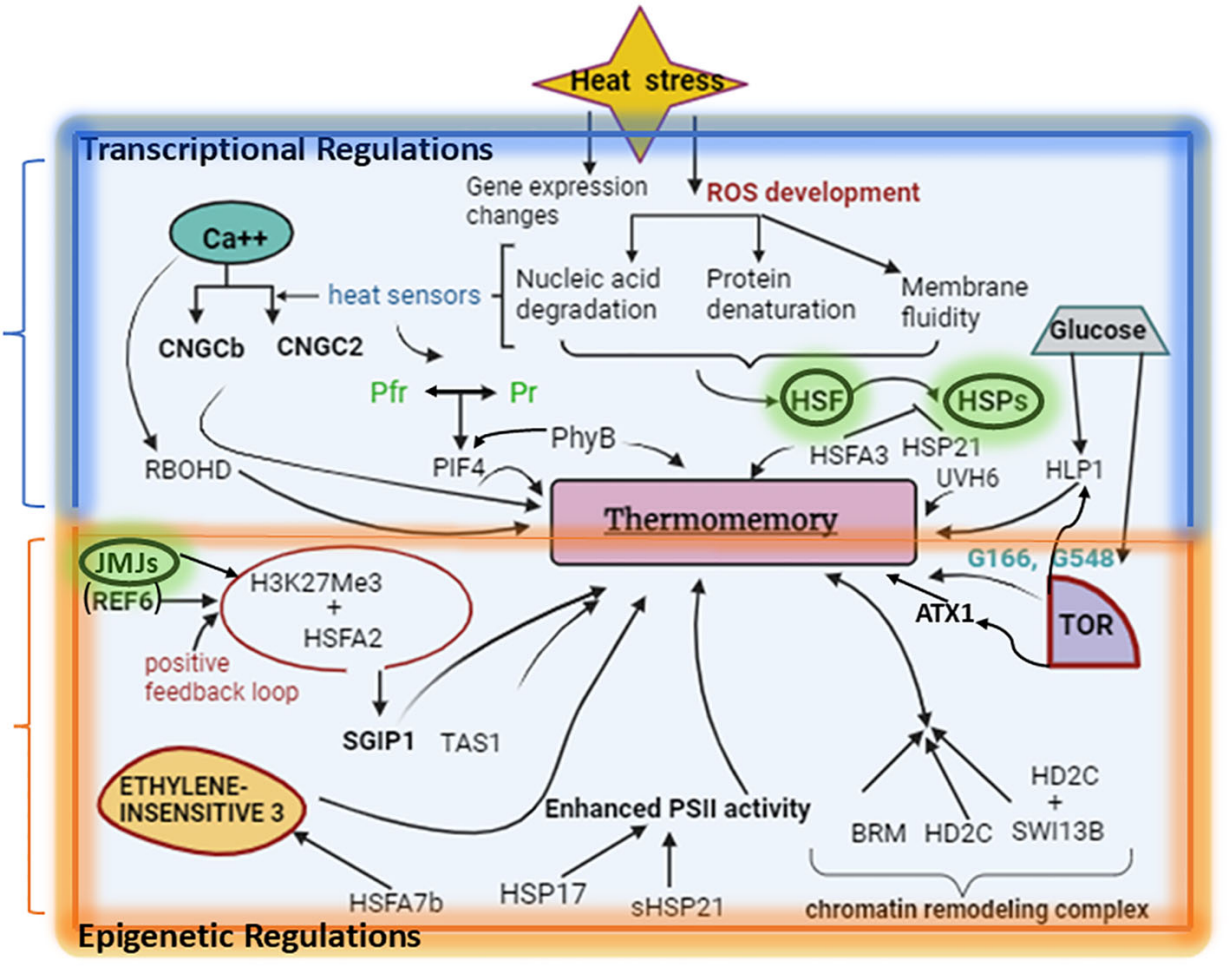
Generation of Thermomemory in Plants. When there is heat stress, the accumulation of ROS results in nucleic acid degradation, protein denaturation, membrane fluidity, and activation of HSPs and HSFs. Key components of thermotolerance are HSFs and HSPs. When the environment becomes stressful, the membrane-localized RBOHD produces apoplastic ROS, which in turn triggers HSFs indirectly. The calcium released from the plasma membrane and endoplasmic reticulum via CNGC- and IP3-gated calcium channels, which permit an influx of calcium into the cytoplasm, activates the CDPK, which controls RBOHD. The generation of splice-variants (HsfA2-III) of HsfA2, which is crucial for thermomemory, is caused by alternative splicing that is induced by HS. Thermotolerant plants have enhanced expression of many HSFs, HSPs, and genes for increased osmolyte levels. Nucleus-coded small HSPs, such as sHSP21, play a vital role in safeguarding PS-II and other photosynthesis-related machinery and greatly enhance HS tolerance. A positive feedback loop between HSFA2 and H3K27Me upregulate SGIP1 and TAS1 which favorably regulate thermotolerance. The orange area depicts the epigenetic regulations while the blue area shows transcriptional regulation of thermomemory. Key molecules (HSFs, HSPs, JMJ proteins) are shown in green highlighted oval. This figure was generated using BioRender (www.biorender.com).

Moreover, the master regulators of the heat stress response (HSR) are a group of transcription factors (TFs) known as DNA-binding heat shock transcription factors, or HSFs (Balazadeh, 2022). These transcription factors (TFs) control the activation and subsequent fast synthesis of downstream heat shock proteins (HSPs) balancing cellular integrity by acting as molecular chaperones (**Figure 1**). This shields plants from cellular damage and allows the stabilization of the refolding of heat-inactivated proteins.

It has been reported that there are 20 distinct kinds of HSF genes in Arabidopsis. Protein-wise, HSFs are made up of two conserved domains: HR-A and HR-B, which are hydrophobic heptad domains essential for oligomerization, and a conserved DNA-binding domain (DBD) in the N-terminal that can recognize Heat Shock Elements (HSE) for target recognition. These can be divided into three classes according to the number of amino acids that separate the HR-A and HR-B domains: HSFA, HSFB, and HSFC. According to reports, HSFA1a, HSFA1b, HSFA1d, HSFA1e, HSFA2, and HSFA3 are essential for the development of primary heat response. Among these, DREB2A (Dehydration-Responsive Element-Binding 2A) is a downstream transcription factor that HSFA1 directly targets. DREB2A then activates HSFA3 to trigger HSR for a prolonged duration of survival (Balazadeh, 2022). At the same time, HSFA2 binds to HSFA, which activates a chaperone-like protein called Heat Stress Associated 32 (HSA32), which plays a major role in the establishment of HSR and stress-induced memory in primed plants as well as the preservation of cellular homeostasis.

A significant concentration of HSA32 during thermomemory or HSP101 buildup that is ongoing controls the recovery phase. Retardation of HSA32 simultaneously creates a positive feedback loop that keeps HSP101 consistently stable. Depending on their molecular weight and period of upregulation, HSPs can be divided into families, such as HSP101, HSP21, HSP22, HSP17.6, HSP90, HSP70, and HSP18.2 (Balazadeh, 2022).

In developing thermomemory, the expression of these genes is stimulated at various times after exposure to high temperatures. In a study, genes triggered by heat stress, such as HSP101 and HSP70, are expressed immediately in reaction to heat and then gradually decrease when the stress subsides. On the other hand, several genes are increased during the stress-induced memory phase, including HSP18.2, HSP21, and HSP22. It has been documented that the remaining HSP genes express and become activated in response to ensuing exposure to heat (Ling et al., 2018).

## 3 Signaling and regulation during thermomemory

Heat-related memory genes, which are dormant in normal environmental conditions, are transcribed in response to heat shock (HS) to give stability and expression. Two categories of transcriptional memory have been identified based on transcriptional patterns. The transcription of such dormant genes is part of the initial pattern, known as Type I transcriptional memory, and it persists for a few hours or days even after the stress has subsided. However, in response to HS exposure, some genes have a stronger transcriptional response. Since these genes can remember the first heat exposure, their transcription increases significantly when they are exposed to HS again. Type II transcriptional memory is the term used to describe this kind of transcription pattern.

### 3.1 Regulation of type I transcriptional memory

HSFs sustain these two kinds of transcriptional patterns after being exposed to HS. Among the HSF proteins, HSFA2 and HSFA3 work in tandem with histone alterations to primarily mediate Type I transcriptional memory (Nishio et al., 2024). When heat stress occurs, HSFA1 is instantly activated, controlling the expression of HSFA2. In primed plants, the pre-mRNA transcript of HSFA2 experiences alternating splicing, resulting in the shortened splice variant isoform known as HSFA2-III (Ling et al., 2021). To mediate the production of other HSP genes, namely HSP21, HSP22, and HSP18.2, this isoform binds to the HSE of HSFA2. Relative Of Early Flowering 6 (REF6), the gene encoding the H3K27Me3 demethylase enzyme, is directly activated upon HSFA2 expression. Additionally, this REF6 directly contributes to HSFA2’s de-repression. By keeping HSFA2 in a stable “on-state,” The epigenetic heat shock memory is extended via a positive feedback loop that is established by HSFA2 and H3K27Me3 demethylase. This loop also activates the E3 ubiquitin ligase SUPPRESSOR OF GENE SILENCING 3 (SGS3)-INTERACTING PROTEIN 1 (SGIP1), which leads to the eventual upregulation of heat-induced TAS1 target 5 (HTT5) expression, favorably regulating thermotolerance (Szaker et al., 2020).

### 3.2 Regulation of type II transcriptional memory

Similarly, Type II transcriptional memory is regulated by a different group of chromatin modifiers or histone demethylases known as Jumonji family proteins (JMJC), also termed H3K27me3 demethylases. These proteins include JMJ30, JMJ32, JMJ13, JMJ12 (also known as REF6), and JMJ11 (also known as Early Flowering 6, ELF6) (Nishio et al., 2024). Although the physiological roles of these five demethylases are identical, their identification of target genes varies. JMJ13, REF6, and ELF6 are the three members of the same plant-specific KDM4 subfamily. The zinc finger (ZnF) domains C2-H2 type make up REF6, which can detect double-stranded DNA at the target gene sequence that is encoded with the CTCTGYTY motif. Since ELF6 has also been shown to have C_2_-H_2_ type ZnF domains, the CTCTGYTY motif may interact with ELF6’s DNA recognition motif. JMJ13 differs from ELF6 and REF6 in that it lacks ZnF domains but instead uses a catalytic domain to engage with the H3K27me3 peptide in its DNA recognition mechanism. Furthermore, JMJ30 is recognized as the first demethylase to primarily express itself in response to heat stress. It accomplishes this by binding to the HSP16.6, HSP21, and HSP22 gene loci and eliminating H3K27me3 (Yamaguchi et al., 2021). H3K27me3 levels are kept lower during the memory phase even after heat stress passes and ambient temperature returns to normal. The second heat shock exposure comes next, during which JMJ30 reinstates the expression of these genes. Furthermore, JMJ32, working in tandem with JMJ30, controls the elimination of H3K27me3 from thermomemory-related HSP17.6 and HSP21, aiding in the recovery.

Furthermore, A study conducted in Arabidopsis reported that an essential element of thermomemory is HSP21, a plastidial small heat shock protein that quickly builds up upon heat stress and is abundant during the thermomemory phase. Maintaining high levels of HSP21 is necessary for sustained memory. They demonstrated that HSP21 abundance is regulated by the plastid-localized metalloprotease FtsH6, using transcriptome profiling and pharmacological interrogation. The potential for thermomemory is increased and HSP21 accumulation is encouraged in the later stages of thermomemory when the FtsH6 protein is not functioning (Sedaghatmehr et al., 2016). Glucose is also essential to produce thermomemory in Arabidopsis because it regulates HIKESHI-LIKE PROTEIN1 (HLP1). The regulation of this protein is responsible for conferring thermotolerance in transgenic Arabidopsis plants (Sharma et al., 2019). According to a new study, heat priming wheat during the stem elongation stage greatly reduces the harm that HS causes to grain yield during grain filling (Wang et al., 2016). Another research revealed that establishing Plant Stress Memory (PSM) and promoting improved adaptation in subsequent stages are achieved by priming plants with mild primary stress and priming agents, whether biological or pharmacological. This method has prospects as an approach to develop climate-resilient crops (Siddique et al., 2024).

Thermomemory not only showcases the plasticity of plant responses to environmental challenges but also hints at the existence of intricate memory-like systems within the plant kingdom. It has been revealed recently that in Arabidopsis plants, thermopriming activates the splicing memory (Ling et al., 2018). Changes in the expression of thermomemory genes between the first and subsequent exposures are linked to epigenetic alterations of these genes (Bäurle and Trindade, 2020). Histone modifications are primarily carried out by two types of enzymes: writers and erasers. The increased expression and thermotolerance of the HSFA3 and UVH6 genes in Arabidopsis are linked to GCN5’s improvement of acetylation of H3K9/K14Ac in the promoter region of these genes (Nishad and Nandi, 2021). The protein SW13B of the BRAHMA (BRM), SWI/SNF (SWITCH/SUC NONFERMENTING) complex for chromatin remodeling interacts with the HD2C deacetylase. The three factors that regulate plant thermotolerance are BRM, HD2C, and SWI13B (Brzezinka et al., 2016; Buszewicz et al., 2016).

Thermotolerant plants have upregulated the expression of many genes, HSFs, and HSPs for increased osmolyte levels. Some research discovered that increased resistance to HS is mediated by Glc-regulated Arabidopsis TOR. Tor 35-7 RNAi plants showed a reduced response to thermotolerance, but Arabidopsis plants that overexpressed TOR (G166 and G548) showed enhanced thermotolerance (Sharma et al., 2019). According to them, the contrasting traits in stress responses could be the result of variations in stress and recovery conditions. For example, when plants recovered after HS, Higher levels of Glc/TOR at that time activated growth signaling, as both the TOR G166 and G548 lines showed improved stress mitigation (Sharma et al., 2019).

According to a different study, autophagy, a key mechanism of self-degradation, helps Arabidopsis cells refresh their memory of heat stress (HS). Plants are protected from high temperatures by HS, which increases the development of autophagosomes and the expression of genes relevant to autophagy. Thermopriming (moderate HS) induces autophagy, which interestingly stays elevated even after stress cessation. They show that, later in the thermorecovery phase, autophagy mediates the selective destruction of heat shock proteins, which results in the formation of protein aggregates following the second heat shock and a reduced ability to withstand heat. Autophagy mutants exhibit enhanced thermomemory while retaining heat shock proteins longer than the natural type (Pu et al., 2017).

A study reported that HEAT SHOCK TRANSCRIPTION FACTOR A7b of HSFA7b is essential to this process in Arabidopsis since the reduction of SAM activity over time that occurs during thermopriming is caused by the lack of functional HSFA7b. It was discovered that HSFA7b, which binds it directly regulates the ethylene response at the SAM of the key ethylene signaling gene ETHYLENE-INSENSITIVE 3, thereby establishing thermotolerance (John et al., 2023). Another study stated that in tall fescue, overexpression of FaHSP17.8-CII was found to increase PSII activity and chlorophyll content under ACC+HS compared to HS alone and to decrease ROS formation and chloroplast ultra-structure damage. These results identify a transcriptional memory-regulating FaHSP17.8-CII-PSII-ROS module that improves thermotolerance in cool-season turfgrass (Bi et al., 2021). Small HSPs with nucleus coding, such as sHSP21, are essential for safeguarding PS-II and other photosynthesis-related equipment and greatly enhance HS tolerance (Bi et al., 2021).

Plant thermomemory is a complex network of epigenetic and molecular pathways that allow them to adapt to heat stress (HS). Heat-induced transcriptional memory genes are divided into two types: Type I, which show temporary activation, and Type II, which show improved responses after repeated exposure, members such as HSFA2, HSFA3, and histone modifiers such as REF6 and JMJ family members all play important roles in the regulation of memory processes. Epigenetic alterations, such as H3K27me3 demethylation, help to maintain the memory phase, while heat shock proteins (HSPs) like HSP21 give long-term protection. Autophagy, glucose-regulated TOR signaling, and chromatin remodeling all contribute to thermotolerance. These findings highlight the possibility of thermopriming and genetic interventions in creating climate-resilient crops with improved heat stress tolerance. Plant adaptive strategies include genetic modification to increase the expression of HSPs and transcription factors such as HSFA2 and HSFA3, epigenetic priming to maintain transcriptional memory, and the development of thermopriming protocols to precondition crops for future heat stress, creating transgenic crops with enhanced heat tolerance using CRISPR-Cas9, leveraging small HSPs such as HSP21 for photosynthetic machinery protection, and optimizing autophagy have the potential to improve agricultural resilience and sustainability in a warming environment.

## 4 Role of nitric oxide under heat stress and thermomemory

Before delving into the specifics of thermomemory, it is crucial to appreciate the multifaceted role of NO as a signaling molecule in plants. From its participation in stomatal regulation to its influence on gene expression, NO acts as a molecular conductor orchestrating responses to various environmental stimuli. Nitric oxide (NO), a gaseous free radical, stands as a sentinel in the intricate landscape of cellular communication within plant physiology. Operating as a versatile and ubiquitous signaling molecule, NO transcends traditional roles, exerting influence over a myriad of physiological processes with profound implications for plant growth, development, and responses to environmental stimuli (Pande et al., 2022a; Khan et al., 2023). Generated enzymatically through the activity of nitric oxide synthase (NOS)-like enzymes or non-enzymatically via the reduction of nitrate and nitrite, NO operates as a dynamic player of diverse signaling pathways (Pande et al., 2022b). Its significance spans from the modulation of stomatal aperture and regulation of vascular development to the mediation of defense responses against pathogens and participation in abiotic stress responses, including those triggered by heat stress (Förstermann and Sessa, 2012).

Within the intricate network of signaling cascades, NO often operates in tandem with other signaling molecules such as reactive oxygen species (ROS), calcium ions (Ca²^+^), phytohormones (e.g., abscisic acid, salicylic acid, jasmonic acid, and ethylene), and secondary messengers like cGMP and cADPR, contributing to the regulation of cellular responses. Furthermore, NO’s role extends to the realm of gene expression, where it participates in the activation or repression of specific genes, thus influencing the transcriptional environment. As an essential mediator of intercellular and intracellular signaling events, NO unveils a fascinating signaling molecule in the plant system, providing researchers with a more thorough understanding of the intricate regulatory networks that govern plant life (Khan et al., 2023). Understanding the intricate roles of NO in stomatal regulation provides valuable insights into the broader mechanisms of plant adaptation to environmental fluctuations, contributing to the optimization of water use efficiency and overall stress resilience (Wilson et al., 2009).

### 4.1 NO-mediated regulation of heat stress

Stomatal closure is a common response to heat stress in plants, helping to reduce water loss. NO is a signaling molecule that participates in the regulation of stomatal closure, particularly in response to various environmental stressors. When plants experience stress, elevated levels of NO are often observed, contributing to the initiation of signaling cascades that result in stomatal closure (Lau et al., 2021). This closure helps reduce water loss, conserving precious water resources during challenging conditions. NO interacts with ABA signaling pathways and studies suggest that NO may act downstream of ABA in mediating stomatal closure. This crosstalk between NO and ABA highlights the intricate integration of multiple signaling pathways in the regulation of stomatal dynamics (Sun et al., 2019). The movements of stomata are primarily controlled through modifications in turgor pressure within specialized guard cells surrounding the stomatal pore. NO is known to influence guard cell signaling pathways, leading to alterations in ion fluxes and water movement. This, in turn, modulates turgor pressure and contributes to the regulation of stomatal aperture.

NO responds to a variety of environmental stimuli, including light, temperature, and pathogen attack (Kashtoh and Baek, 2021). For instance, NO production can be triggered by changes in light intensity, influencing stomatal opening during photosynthesis. The balance between NO and Reactive oxygen species (ROS), including hydrogen peroxide and superoxide, is crucial in stomatal regulation. NO can modulate ROS levels in guard cells, which regulates stomatal movements. This interplay helps plants adjust stomatal aperture in response to both abiotic and biotic stresses (Singh et al., 2017). Moreover, reactive nitrogen species are produced when NO interacts with molecular oxygen and/or O_2_-anion (RNS). Together, these molecules transduce signals under stressful situations and keep the redox balance within the cell and is crucial for priming techniques that improve cross-tolerance (Khan et al., 2022).

Wodala et al. (2008) reported that NO has been shown to induce the expression of stress-responsive genes. These genes often encode proteins that are involved in the plant’s defense mechanisms against environmental stresses, such as pathogen attack, drought, salinity, and high temperatures. High temperature induced NOS and GSNOR activities, which were followed by the accumulation of NO and S-nitrosothiols, without significantly altering the content of nitrites and nitrates in P. sativum leaves. This suggests that NOS activity is the cause of the NO produced in P. sativum leaves (Wodala et al., 2008). Understanding NO-mediated responses to heat stress not only improves our understanding of plant stress physiology, but it also has implications for developing targeted strategies such as engineering NO signaling pathways, modulating antioxidant systems to reduce ROS, increasing heat shock protein (HSP) expression, and incorporating NO donors or priming agents to improve crop thermotolerance. These approaches have the potential to greatly contribute to long-term agricultural sustainability in the face of climate change (Park and Seo, 2015).

### 4.2 NO-mediated regulation of thermomemory

**Figure 2:**
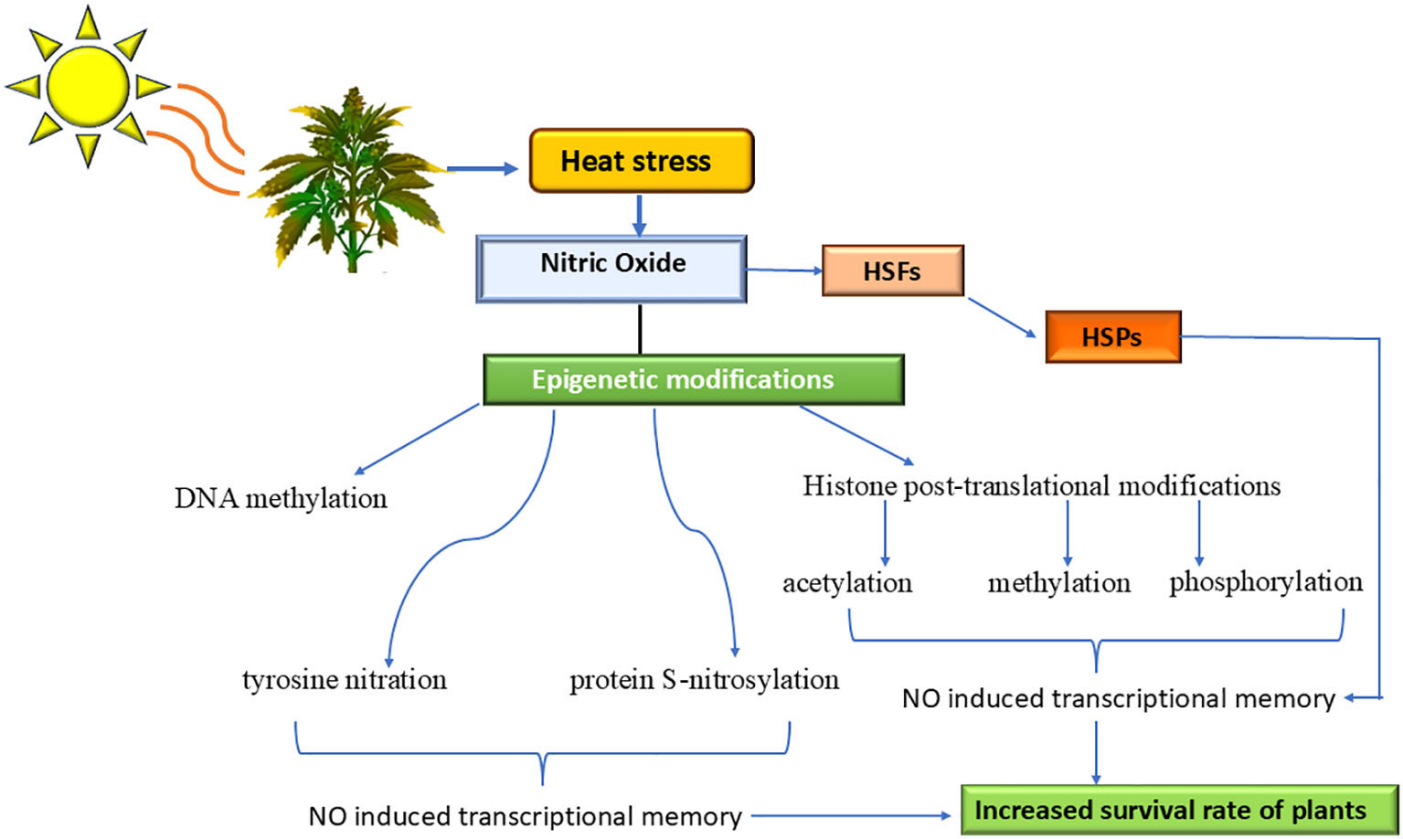
Role of Nitric Oxide in thermomemory in Plants. Nitric oxide is accumulated in response to heat stress, activating multiple transcriptional factors, including HSF, which in turn increases the expression of HSPs. NO-induces thermomemory in plants through different epigenetic regulations and protect the plants from the stressful environment.

It has been reported that NO has a role in thermotolerance. NO and its derivatives modify gene expression at the transcriptome and proteome levels by causing post-translational changes such as nitration, S-nitrosylation, and binding to tyrosine residues in DNA, proteins, lipids, and thiols (**Figure 2**). It has been reported that the buildup of NO and its derivatives following the administration of heat stress is what triggers the expression of HSFs and HSPs. Xuan et al. (2010) observed that NO production starts 10 minutes after 45°C heat shock to Arabidopsis plants and then increases until it reaches its maximum between 60 and 70 minutes of heat shock. This process has been reported to be regulated by HSP18.2. They identified evidence of the involvement of NO in thermotolerance and concluded that NO positively influences thermotolerance in Arabidopsis by increasing the DNA-binding activity of heat shock transcription factors (HSFs) and the accumulation of HSP18.2.

NO can modulate gene expression in response to various environmental cues. It may influence the expression of genes associated with heat shock proteins and other protective proteins involved in thermotolerance. One study reported that through acting downstream of ROS, nitric oxide (NO) is a newly identified signaling molecule in the heat stress response. Increased production of nitric oxide and ROS has also been linked to increased thermotolerance (Seth and Sebastian, 2024). When exposed to heat stress, Arabidopsis seedlings with specific RBOH mutations produce less NO than WT plants and exhibit greater heat sensitivity. With NO donations, this can be avoided. NO appears to activate antioxidant enzymes and control the overproduction of ROS during stress. Important signaling proteins like CDPKs, CaM, and HSPs are also S-nitrosylated by NO. It was hypothesized that S-nitrosylation plays a crucial role in the heat stress response pathway in plant thermotolerance because it alters their stability, location, and activity (Dündar et al., 2024; Mariana Distéfano et al., 2024). In cool-season turfgrasses, it was shown that gas molecules such as carbon dioxide and nitric oxide had protective effects on heat stress tolerance (Sun et al., 2024).

Lee et al. (2008) reported that nitric oxide (NO) serves as a versatile signaling molecule that can modulate gene expression in response to a wide array of environmental stresses in plants. According to their findings, null mutants of hot5, which codes for S-nitrosoglutathione reductase, had higher amounts of nitrate and nitroso species, and both missense and null alleles’ heat sensitivity was linked to higher levels of NO species. NO donors improved the heat sensitivity of both wild-type and mutant plants, and a NO scavenger was able to successfully restore the heat sensitivity of the hot5 mutants. Therefore, there study suggested that heat sensitivity in Arabidopsis was caused by an increase in NO levels. Xuan et al. (2010) suggested that, NO act as secondary messenger and helps AtCaM3 induce thermotolerance, which is reliant on increasing the activity of HSF DNA binding and accumulation of HSP. They proposed that NO homeostasis is a critical component influencing the beginning of resistive responses to elevated temperatures, which states that a high NO level reduces thermotolerance. According to Guo et al. (2024) when Ca^2+^-CAM binds to S-nitrosoglutathione reductase, it inhibits its activity, increasing intracellular nitric oxide levels and improves plants’ ability to withstand heat.

Tomato plants with enhanced thermotolerance benefit from exogenous NO administration as it neutralizes ROS and produces dehydroascorbate. The process of osmo-priming during seed germination leads to enhanced germination by repressing the antioxidant pathway that involves catalase and superoxide dismutase (SOD) and activating the ascorbate peroxidase (APX) scavenging mechanism (Nair et al., 2022).

NO becomes a key component in optimizing the plant’s resistance to heat shock and its capacity to remember previous experiences. A sophisticated and intricately coordinated group of cellular processes within plant physiology is revealed by the molecular kinetics of NO-mediated responses to heat stress. Heat stress disrupts cellular homeostasis, and NO is a major modulator that affects several molecules that are essential for thermotolerance. Notably, NO is implicated in the activation of heat shock proteins (HSPs), a family of chaperones pivotal in protein folding and stability under stress conditions. Furthermore, NO participates in the regulation of antioxidant defenses, playing a crucial role in mitigating oxidative stress induced by elevated temperatures. The intricate crosstalk between NO and these molecular components extends to pathways involved in stomatal regulation, gene expression, and hormonal signaling, collectively shaping the plant’s response to heat stress.

NO can activate specific transcription factors that act as master regulators of stress-responsive genes. Transcription factors bind to the promoter areas of target genes, initiating or enhancing their transcription. It was reported that NO mediates HSFA2 which directly activates H3K27me3 demethylase REF6, hence derepressing HSFA2. REF6 and HSFA2 activate SGS3-SGIP1 resulting in a heritable feedback loop for regulating thermomemory. Trans-acting siRNA (tasiRNA) production is suppressed as a result of SGIP1-mediated SGS3 degradation. When the REF6-HSFA2 loop and reduced tasiRNA converge, HEAT-INDUCED TAS1 TARGET 5 (HTT5) is produced, which promotes early blooming but lowers immunity. Therefore, heat induces transmitted phenotypes through a coordinated epigenetic network comprising transcription factors, tasiRNAs, and histone demethylases, ensuring both reproductive success and transgenerational stress adaptation (Wang et al., 2023). According to a study, epigenetic changes may play a part in the formation of a “Thermomemory,” which raises the possibility that these modifications can influence how plants’ innate immunity is regulated when exposed to stress (Rai et al., 2020).

Previous research has shown that heat stress raises the amount of NO in wheat, and that exogenous NO administration increases thermotolerance in wheat and Lablab purpureus (Rai et al., 2018). Significant variance has been observed in studies on NO-mediated thermotolerance, and the connection between NO production and stress resistance is not fully understood. H_2_O_2_-dependent NO synthesis has been discovered to be caused by abscisic acid (ABA), while endogenous NO generation is required for ABA and H_2_O_2_-mediated increases in MAPK and antioxidant gene expression. ABA is necessary for NO-mediated thermotolerance. Through upregulating gene expression and resulting in the production of small HSP26, NO shields chloroplasts from oxidative damage during heat stress. The regulation of HSP70 synthesis and accumulation under heat stress has been demonstrated to involve NO. Previous studies have shown that NO, whether exogenous or endogenous, can greatly improve plant thermotolerance. It has been proven that NO greatly boosts *Vicia faba* plants’ thermotolerance (Khan et al., 2023). It has been demonstrated that raising NO levels in plants increases their tolerance to heat stress, making it a viable target for the development of strategies aimed at lessening the detrimental effects of heat stress on plant growth and productivity. According to a study, H_2_S may enhance maize thermotolerance as a signaling molecule. This is related to the signaling crosstalk between H_2_S and nitric oxide (Li et al., 2024).

## 5 Nitric oxide mediated epigenetic regulations

Majority of the studies done on thermomemory in plants in the past 20 years are linked to epigenetic modifications as detailed by Nishad and Nandi, 2021. Systemic acquired resistance (SAR) is likely caused by epigenetic changes of HSFs. Thermomemory and stress priming are linked to genetic and epigenetic control, particularly of HSPs and HSFs. According to recent research, in plants such as Arabidopsis (Suter and Widmer, 2013), Wheat (Migicovsky et al., 2014), Rapeseed (Byeon et al., 2019), and others (Liu et al., 2020) HS induces heritable phenotypic and epigenetic alterations. NO has been implicated in inducing epigenetic modifications, such as DNA methylation and histone modifications (**Table 1**). These modifications can alter the accessibility of genes, influencing their expression patterns. The epigenetic regulation by NO provides a long-term memory of stress exposure (Socco et al., 2017).

**Table 1:** List of epigenetically regulated genes and biomarkers modulated by nitric oxide under heat stress to confer thermotolerance in plants.

S.No.	Epigenetically regulated genes and biomarkers under heat stress	Response	Plant species	Reference
**1**	NADPH oxidase	NO works downstream of H_2_O_2_ in signaling.	*Arabidopsis thaliana*	Wang et al., 2014
**2**	trHb	Chemical scavengers for reactive nitrogen species may enhance seed germination at high temperatures.	*Arabidopsis thaliana*	Hossain et al., 2010
**3**	Arginine amidohydrolase-1 and -2	Increased NO buildup compared to the wild-type.	*Arabidopsis thaliana*	Flores et al., 2008
**4**	Nitrate reductase	NO plays a role in signaling, stomatal closure, and acts upstream to AtCaM3 activation.	*Arabidopsis thaliana*	Xuan et al., 2010
**5**	Chloroplast phosphoenolpyruvate/phosphate translocator	NO induces Antioxidant and osmolyte levels	*Arabidopsis thaliana*	He et al., 2004
**6**	Silenced Atgsnor1-3	Nitrate concentrations increased	*Arabidopsis thaliana*	Fröhlich and Durner, 2011
**7**	S-nitrosoglutathaione reductase	Normal plant growth, fertility, and temperature tolerance depend on GSNOR-regulated NO homeostasis and GSNOR function.	*Arabidopsis thaliana*	Lee et al., 2008
**8**	Nitric oxide associated1-suppressed RNAi lines	Temperature-dependent control of rice plastid growth, rubisco generation, and chlorophyll biosynthesis	*Oryza sativa*	Yang et al., 2011
**9**	AtNIA2-overexpressing transgenic lines	H_2_O_2_ acts upstream of NO in thermotolerance, increasing the seedlings’ survival ratio.	*Arabidopsis thaliana*	Wang et al., 2014
**10**	Transformed guard cell protoplasts with auxin responsive BA promotor	Heat stress can still harm plants that have adapted to tolerate prolonged high temperatures.	*Nicotiana glauca*	Beard et al., 2012
**11**	Tyrosine nitration and protein S-nitrosylation	NO provide resilience to stress	*Arabidopsis thaliana*	Corpas et al., 2015
**12**	Reduced H_2_O_2_ and MDA	NO increased the survival	*Oryza sativa*	Song et al., 2013
**13**	Decreased H_2_O_2_ and MDA and increased proline	NO showed tolerance towards heat stress.	*Zea mays*	Kaur and Kaur, 2018
**14**	Increased Antioxidant activity (SOD, CAT, APX, GR), heat shock proteins	NO regulates thermotolerance	*Vicia faba*	Alamri et al., 2019
**15**	Exogenous ABA treatment resulted in further increase of NOS activity	NO act as an intermediate molecular mediating abscisic acid induced thermotolerance under heat stress.	*Phragmites communis*	Song et al., 2008
**16**	AtCaM3 and *S*-nitrosoglutathione reductase (GSNOR)	Inducing an increased GSNOR activity and an inhibited NO level regulate thermotolerance	*Arabidopsis thaliana*	Zhang et al., 2020
**17**	CNGC6	Acts upstream of NO in the HS pathway, which improves thermotolerance	*Arabidopsis thaliana*	Peng et al., 2019
**18**	RESPIRATORY BURST OXIDASE HOMOLOG1 (SlRBOH1)	In responses to elevated temperatures, GSNOR regulates the SlRBOH1-dependent apoplastic H_2_O_2_ generation.	*Solanum lycopersicum*	Song et al., 2022

NO affects a variety of epigenetic regulators, including structural DNA binding proteins and histone changes. Furthermore, NO can induce gene expression, inflammation, genomic instability, carcinogenesis, and dysregulate DNA methylation and acetylation. Recent years have seen significant advancements in our knowledge of the post-translational modifications (PTMs) caused by NO in plants, such as tyrosine nitration and protein S-nitrosylation, which have a profound impact on the activity of numerous enzymes that provide resilience to stress (Corpas et al., 2015). Gene expression modifications that are heritable and reversible without involving changes to DNA sequence are known as epigenetic regulation (Socco et al., 2017). The most common epigenetic regulators are DNA methylation, histone post-translational modifications (acetylation, methylation, or phosphorylation), and non-coding RNAs (ncRNAs) associated with the altered pattern of mRNA translation. Various research indicates that NO and miRNA expression are closely related. Furthermore, miRNA expression is widely affected by lncRNAs, which inhibits the downstream processes of miRNAs (Li et al., 2024). The activity of KDM3A (Jumonji domain-containing 1A) demethylase and histone 3 have been demonstrated to be inhibited by NO, which changes the methylation state and causes an accumulation of the substrate of histone H3K9me2 (Hickok et al., 2013).

It was reported that the demethylases KDM3B, KDM4A, KDM4B, KDM4C, and KDM4D were up-regulated, particularly KDM7A and KDM1, to counteract this inhibition. According to functional investigations, by entering the catalytic site of the demethylase directly, NO creates a nitrosyl–iron complex (Hickok et al., 2013). In a CpG dinucleotide, a methyl group is covalently bonded to the fifth carbon of the cytosine pyrimidine ring, resulting in DNA methylation, an epigenetic alteration (Hardy and Mann, 2016). In a study, the enzymatic reaction is mediated by three different DNA methyltransferases (DNMT1, DNMT3a, and DNMT3b), which use S-adenosyl-methionine as a methyl donor (Tsai and Baylin, 2011). When NO increases DNMTs’ posttranscriptional activity, CpG island methylation accumulates and gene expression is suppressed (Hmadcha et al., 1999). According to a recent study, 22-nt siRNAs are crucial for plant adaptability and thermotolerance. 22-nt siRNAs produced by NIA1/2 transcripts cause translational repression of these transcripts in a nitrogen-deficient environment, which lowers amino acid levels, reduces global translation, and causes a shift from growth to defense (Pan et al., 2024).

Another study found that NO was sensitive to HDA6 activity, indicating that NO regulates histone acetylation. RNA-seq and chromatin immunoprecipitation sequencing results also showed that NO is involved in the metabolic transition from growth and development to stress response (Ageeva-Kieferle et al., 2021). According to a different study, NO inhibits and targets HDAC complexes to affect histone acetylation, which causes some genes to become hyperacetylated. This method mediated the transcription of genes triggered by stress in plants during their stress response (Mengel et al., 2017). Additional research on Arabidopsis reveals a mechanism by which plants respond to environmental changes by converting stress-induced NO signal to protein methylation machinery via S-nitrosylation of PRMT5 (Hu et al., 2017: Pande et al., 2022b).

## 6 Conclusion

The concept of thermomemory, wherein plants exhibit a capacity to ‘remember’ and adapt to previous heat stress events, adds a degree of intricacy to our understanding of plant resilience. This review explores and highlights our current understanding of heat stress responses in plants particularly the molecular pathways that are activated as a mechanism to cope with the heat stress in plants. Further broadening our knowledge about the complex role of NO in heat stress as well as thermomemory development. From the activation of heat shock proteins to the regulation of antioxidant defenses, NO emerges as a central player in regulating the plant’s ability to withstand thermal challenges and retain information about past encounters.

## 7 Future prospects

The review projects the importance and the unexplored roles of NO in mediating thermomemory in plants. Exploring the signaling events and targeting the key players in thermomemory could revolutionize crops to enhance productivity under heat-stress conditions. For example, in wheat (*Triticum aestivum*), exogenous NO donors have been found to improve thermotolerance by stabilizing photosynthetic machinery during heat stress. In rice (*Oryza sativa*), NO regulates heat shock protein production, which improves grain yield under high-temperature circumstances. Tomato (*Solanum lycopersicum*) is more resistant to heat stress when NO modifies antioxidant activity and reduces oxidative damage. Furthermore, in *Arabidopsis thaliana*, NO has been intensively researched for its function in thermomemory by modulating transcriptional memory genes such as HSFA2. These examples demonstrate the potential of using NO signaling to improve thermotolerance in a variety of crops, paving the path for sustainable agriculture.

## Glossary

AtCaM3: Arabidopsis thaliana calmodulin 3
APX: Ascorbate peroxidase
BRM: Brahma
CDPK: Calcium-dependent protein kinases
CNGC: Cyclic nucleotide-gated calcium channel
DBD: DNA-binding domain
DREB2A: Dehydration-Responsive Element-Binding 2A
DNMT: DNA methyltransferases
GCN5: General control nonderepressible5
H3K9me2: H3 lysine 9 dimethylation
HATs: Histone acetyltransferase
HD2C: Histone deacetylases 2C
HDACs: Histone deacetylases
HDMs: Histone demethylase
HS: Heat stress
HLP1: Hikeshi-like proteins 1
HMTs: Histone methyl transferases
HSA32: Heat Stress Associated 32
HSE: Heat shock element
HSF: Heat shock transcription factor
HSP: Heat shock proteins
HSR: Heat stress response
HTT5: Heat inducible Tas1 target 5
IP3: Inositol-1,4,5-triphosphate
KDM: Lysine demethylase
lncRNA: Long non-coding RNA
MGDG: Monogalactosyldiacylglycerol
NAD+: Nicotinamide adenine diphosphate
NO: Nitric Oxide
NOS: Nitric oxide synthase
PhyB: Phytochrome B
PIF4: Phytochrome interacting factor 4
PIP2: Phosphatidyl-4,5-inositol bisphosphate
PIPK: Phosphatidylinositol phosphate kinase
PLD: Phospholipase D
PRMT5: Protein arginine methyltransferase 5
PS-II: Photosystem-II
RBOHD: Respiratory burst oxidase homolog
REF6: Relative of early flowering 6
RNS: Reactive nitrogen species
ROS: Reactive oxygen species
RSS: Reactive sulfur species
RONSS: Reactive oxygen, nitrogen, and sulfur species
SAR: Systemic acquired resistance
SOD: Superoxide dismutase
sHSP: smaller heat shock proteins
SGIP1: SGS3 interacting proteins
SGS3: Suppressor of gene silencing 3
SWI/SNF: Switch/Suc non-fermenting
tasiRNA: Trans-acting siRNA
TAT: Thermal acquired tolerance
TFs: Transcription factors
UVH6: Ultraviolet hypersensitivity6
